# The acceptability and feasibility of a randomised controlled trial of serial prophylactic exchange blood transfusion versus usual care in pregnant women with sickle cell disease: a qualitative study

**DOI:** 10.1186/s12884-026-09244-5

**Published:** 2026-07-03

**Authors:** Laura L. Oakley, Clare McDermott, Vicky Robinson, Jeannine Joseph, Eugene Oteng-Nim, Sarah B. Brien

**Affiliations:** 1 Department of Non-Communicable Disease Epidemiology, London School of Hygiene & Tropical Medicine, London, UK; 2 Centre for Fertility and Health, Norwegian Institute of Public Health, Oslo, Norway; 3 Faculty of Medicine, Primary Care, Population Sciences and Medical Education, University of Southampton, Aldermoor Health Centre, Aldermoor Close, Southampton, UK; 4 Guy’s and St Thomas’ National Health Service Foundation Trust, London, UK; 5 Division of Women’s Health, Kings College London, St Thomas’ Hospital, London, UK; 6 Faculty of Medicine, Primary Care, Population Sciences and Medical Education, University of Southampton, Southampton General Hospital, Southampton, UK

**Keywords:** Sickle cell disease, Pregnancy, Serial prophylactic exchange blood transfusions, Qualitative, Feasibility trial, Recruitment barriers, Patient decision-making, Treatment preferences

## Abstract

**Background:**

Pregnancy in women living with sickle cell disease (SCD) is associated with increased risk of morbidity and mortality for mother and baby. Outside of pregnancy, serial prophylactic exchange blood transfusion (SPEBT) has proven efficacy as a treatment for acute SCD complications, however, there is inadequate evidence for the safety and benefit for SCD during pregnancy.

**Aim:**

To explore health professionals and women’s view on the acceptability and feasibility of a randomised control trial of SPEBT versus usual care in pregnant women with SCD (Transfusion Antenatally in Pregnant women with SCD—TAPS2).

**Methods:**

Semi-structured telephone interviews were conducted with TAPS2 trial participants, trial decliners and clinical staff working on the TAPS2 trial. Interviews were analysed using reflexive thematic analysis.

**Results:**

We interviewed 19 trial participants, 12 trial decliners and 15 clinical staff. Three overarching themes were identified. First, factors affecting patient decisions on participation. These included personal benefit, particularly the hope that SPEBT would lead to a healthier pregnancy—and altruistic motivations to help other women with SCD. Factors deterring women from participating included concerns about potential risks of SPEBT, for example infection, side effects, or transfusion reactions. Second, experiences of the TAPS2 trial varied. Participants allocated to SPEBT recounted perceived health benefits as well as ‘expected’ side effects. The time and cost involved in attending transfusion sessions had been difficult for some. Clinical staff at some sites reported that pressures on local apheresis capacity made delivering the intervention challenging. Third, interviews provided recommendations to inform the design of a future definitive RCT of SPEBT versus usual care, including suggestions to maximise participant recruitment. Suggested strategies included the provision of accessible, evidenced-based patient education to patients, partners and families to enable informed decision-making. There was strong support for including an observational arm in any future trial to enable patients unwilling, or unable, to be randomised to take part.

**Conclusion:**

Patients’ views on blood transfusions emerged as a key determinant in whether they chose to take part in this feasibility trial. Addressing pregnant SCD women’s treatment concerns and preferences is key to maximising recruitment and retention in future trials. Addressing information gaps or misinformation through accessible patient education is needed. Including an observational study arm as part of a future definitive RCT, along with other highlighted strategies, will ensure that the evidence base and understanding of clinical outcomes in pregnant women with SCD is maximised for future research.

**Trial registration:**

NIH registry (www.clinicaltrials.gov), registration number NCT03975894 (registered 05/06/19); ISRCTN (www.isrctn.com), registration number ISRCTN52684446 (retrospectively registered 02/08/19).

**Supplementary Information:**

The online version contains supplementary material available at https://doi.org/10.1186/s12884-026-09244-5.

## Background

Sickle cell disease (SCD) is the most common inherited disease worldwide, characterised by anaemia, intermittent painful vaso-occlusive episodes (commonly known as ‘crises’) and chronic complications including chronic lung disease, sickle renal disease, stroke and pulmonary hypertension [1]. In sickle cell disease (SCD), an abnormal form of haemoglobin (haemoglobin S, more commonly known as ‘sickle’ Hb) causes red blood cells to become hard, sticky, and curved into a characteristic ‘sickle’ shape, reducing their ability to carry oxygen effectively around the body. This causes the associated symptoms.

In high income and some middle-income countries, the majority of children born with SCD will survive to adulthood [2], and many individuals living with SCD hope to have children of their own [3, 4]. In the UK, approximately 110 pregnancies occur annually in women living with SCD [5]. Pregnancy in women living with SCD is high risk, associated with increased risk of perinatal and maternal morbidity and mortality [6–10] Pregnancy also exacerbates SCD-related complications such as anaemia, painful crisis, pulmonary complications, and infection [5, 10].

There are very limited treatment options for pregnant women with SCD. In the UK, standard care only consists of blood transfusion when clinically indicated [6, 11]. Available current disease-modifying treatments during pregnancy are not recommended [12]. When blood transfusions are clinically required, they can be given as a simple “top-up” transfusion, which, whilst improving oxygen carriage around the body, can potentially cause iron overload and/or fluid overload (hyperviscosity). Alternatively, pregnant women living with SCD can be offered exchange blood transfusion where their own red blood cells (RBC) are removed and replaced with healthy RBC from donors. Exchange transfusions can be done either manually or automatically, using a machine. Where transfusions need to be done on a long-term basis, automated serial prophylactic exchange blood transfusion (SPEBT) is the preferred approach. Exchange transfusions greatly reduce the risks of iron overload and hyperviscosity observed with simple transfusions and can be more effective in lowering the percentage of sickle Hb (haemoglobin S); and therefore are better at reducing the risk of acute SCD complications [13, 14]. All forms of blood transfusion carry a risk of transfusion reactions, including acute and delayed haemolytic reactions, although there is no strong evidence that the risk differs according to the type of transfusion (top-up or exchange). Alloimmunisation (the development of red cell antibodies) is another significant complication of transfusion therapy. While it has been hypothesised that alloimmunisation may be more common with exchange transfusion because of the greater number of red cell units transfused, emerging evidence suggests that the risk is no higher with exchange transfusion compared with top-up transfusion [15–17]. Automated exchange transfusion is resource-intensive and requires specialised equipment and staff with expertise in apheresis procedures.

Outside of pregnancy, serial prophylactic exchange blood transfusion (SPEBT) has proven efficacy as a treatment for acute SCD complications, and for the prevention of pain, acute chest syndrome and strokes in people with SCD [18, 19]. However, there is inadequate evidence for the safety and benefit of SPEBT for SCD during pregnancy [20]. There have been repeated calls for a definitive randomised controlled trial (RCT) to establish the effectiveness of SPEBT in pregnancy [20, 21].

We conducted a multi-centre randomised feasibility trial of SPEBT versus usual care in pregnant women with SCD (TAPS2) to assess the feasibility and acceptability of conducting a definitive RCT of SPEBT in pregnancy. The protocol, analysis plan and feasibility RCT findings have previously been published [22–24]. In TAPS2, participants were randomised to either SPEBT or usual care; with maternal and neonatal outcomes recorded up to 4 weeks post-partum. In summary, 35 participants were recruited (39.8% of those eligible); 18 participants were randomised to intervention, and 17 to usual care, with 100% follow up data achieved. The results confirmed it was feasible to perform a definitive RCT, with satisfactory rates of recruitment and retention. Although not powered to compare outcomes, positive trends in some maternal, infant and postnatal outcomes were identified in the intervention arm.

There is increasing recognition of the importance of conducting qualitative research alongside feasibility trials [25]. Qualitative research within a feasibility trial provides critical insight into why an intervention and/or trial process may or may not work by illuminating participants’ experiences, contextual influences, acceptability issues, and hidden barriers to recruitment, adherence, or implementation barriers that quantitative methods cannot capture. As highlighted by O’Cathain et al and others [26–28], embedding qualitative work in feasibility studies is instrumental in identifying these uncertainties, to strengthen recruitment strategies, optimising trial design and refining intervention or intervention delivery within a definitive trial.

We therefore conducted a qualitative study embedded within TAPS2. The primary aim of this qualitative study was to inform the design for a future definitive trial.

## Methods

An embedded qualitative study was conducted within the multi-centre feasibility RCT assessing SPEBT versus usual care in pregnant women with SCD.

### Study setting and participant recruitment

Participants for the qualitative study were recruited from the same seven NHS hospitals in the UK recruiting patients for the feasibility RCT [24]. We invited three groups of participants involved in the TAPS2 feasibility RCT to participate in the qualitative study: trial participants, women who were eligible for the trial but declined to participate (‘decliners’), and clinicians and research staff involved in delivering the trial. All potential participants were provided with an information sheet and a consent form and the opportunity to ask questions. For context, participants allocated to the SPEBT arm received their first transfusion within 2 weeks of recruitment, and then every 6 to 10 weeks until the end of pregnancy, to maintain HbS% of < 30% or combined HbS and HbC of < 30%. At each transfusion, participants had two cannulas (or drips) inserted to access their veins, which were linked to the apheresis machine: one cannula was be used to remove the blood and the other to transfuse the donor blood (typically between 6–8 units of blood). Cannulas are typically inserted in the patient’s arms, but if venous access was poor, a long line may need to be inserted into a larger vein in the groin, called a femoral line. Trial participants and clinical/research staff who provided written informed consent were purposively sampled to ensure a maximum variation sample across key characteristics (Table 1). We aimed to interview all the decliners who provided informed written consent.

**Table 1:** Sampling characteristics

Trial participants	Staff participants
• Study site • Treatment allocation: intervention/usual care • Trial status: completers/dropouts^*^ • Pregnancy number: 0/≥ 1 • SCD genotype: SS/SC/other • Disease severity: SCD-related hospital admission in last year yes/no • History of transfusion	• Study site • Staff role: clinical role (consultant obstetrician, consultant haematologist)/staff involved in recruitment (research midwife, sickle cell nurse research practitioner)

^*^Dropout status refers to a participant who withdrew from the intervention

Although we prefer the person-centred phrasing “living with sickle cell disease” throughout this manuscript, at times we use the abbreviated form “with SCD” for brevity.

### Data collection

Semi-structured interviews were conducted by telephone (LO and SB) with study participants. Face-to-face interviews were not feasible due to the ongoing Covid pandemic. Informed consent was re-sought verbally prior to the interviews commencing. All interviews were recorded using an encrypted audio recording device.

Interviews with trial participants were conducted at 6–8 weeks post-partum; interviews with decliners were conducted within eight weeks of providing consent; and interviews with trial staff were conducted near the end of, or after, the recruitment phase ended at their site, ensuring participants had adequate time for reflection.

Separate interview topic guides were developed for each participant group by SB and LO, with input from patient contributors and clinicians. Initial interviews for each group were used to pilot and refine the wording of the questions. Consistent with an inductive qualitative approach, new topics arising during interviews were added to the topic guide. Interviews explored the following topics, dependant on the participant group as shown in Table 2.

**Table 2:** Topic questions per participant group

Participant group/ Topic questions	Trial participants	Decliners	Staff
Views on the Acceptability of the trial intervention	✓	✓	✓
Experiences of trial recruitment processes	✓	✓	✓
Reasons for declining participation	N/A	✓	N/A
Experiences of taking part in the trial including randomisation and study conduct	✓	N/A	✓
Experiences of receiving the trial intervention	✓ (Only trial participants randomised to SPEBT)	N/A	N/A
Experience of delivering the trial intervention	N/A	N/A	✓ (relevant clinical staff only)
Views on future research including recommendations for improving recruitment	✓	✓	✓

*N/A* Not applicable

The final topic guides are included as supplementary material [Additional file 1, trial participants; Additional file 2, decliners; and Additional file 3, staff].

Given the study aims, the proposed sample sizes were not aimed to achieve data saturation, rather to elicit a diversity of views through the use of maximum variation sampling approach for staff and trial participants. Purposive sampling, using a maximum variation sampling approach, is a commonly used approach to inform sample size within feasibility RCT qualitative research [25] ensuring an information rich sample to address the study’s aims [29]. Based on our a priori sampling characteristics we aimed to interview 15–25 trial participants; 5–15 decliners; and 15–20 trial staff (two to three staff considered ‘key informants’ in each study site).

The study was conducted by three female post-doctoral researchers (SB, LO and CM), based in medical research departments in two UK universities. CM and SB are primarily qualitative researchers, each with more than 20 years’ experience. LO is primarily a quantitative researcher with more than 15 years’ experience in conducting qualitative research. SB and LO conducted the interviews, and CM led the data analysis with input from SB and LO. All were involved in the write-up. None of the patients were known to the researchers before this study. Some staff interviewees were known to SB and LO prior to interview from earlier stages in the TAPS2 trial, but none were known to CM.

### Analysis

Trial participant and staff interviews were transcribed verbatim, and anonymised transcripts uploaded onto NVivo version 13 for data management and coding (LO, SB and CM). These interviews were analysed by CM using reflexive thematic analysis [30], using the constant comparison approach to compare data within and across individual interviews to identify themes and subthemes.

Trial participant interviews were analysed for themes and subthemes, with additional subgroup analyses to identify any different patterns emerging, including intervention vs control; trial completers vs dropouts (patients who withdrew from the intervention); different illness severity levels; different SCD genotypes; patients with and without prior experience of transfusion; previous pregnancy history and across different trial sites.

Staff interviews were analysed for themes and subthemes, with additional subgroup analysis to compare across study sites [25, 31].

Decliner interviews were not transcribed nor formally analysed, due to funding restrictions. Rather, data was extracted (SB) through repeated listening of the audio files and summarised in tables to report their reasons for declining, suggestions to improve recruitment and study design for the definitive trial. SB manually transcribed selected illustrative quotes across the summary themes, for decliner participants. Illustrative anonymised key quotes are reported. No subgroup analysis was conducted due to limited sample size and data.

Findings across the three participant groups were then mapped onto key themes and sub-themes. Standard methods were employed to ensure rigour (e.g. through the use of audit trail, reflexive diaries, double coding and deviant case analysis). Illustrative anonymised quotes are reported to support themes and subthemes for the data corpus.

## Results

### Participant characteristics

**Fig. 1:**
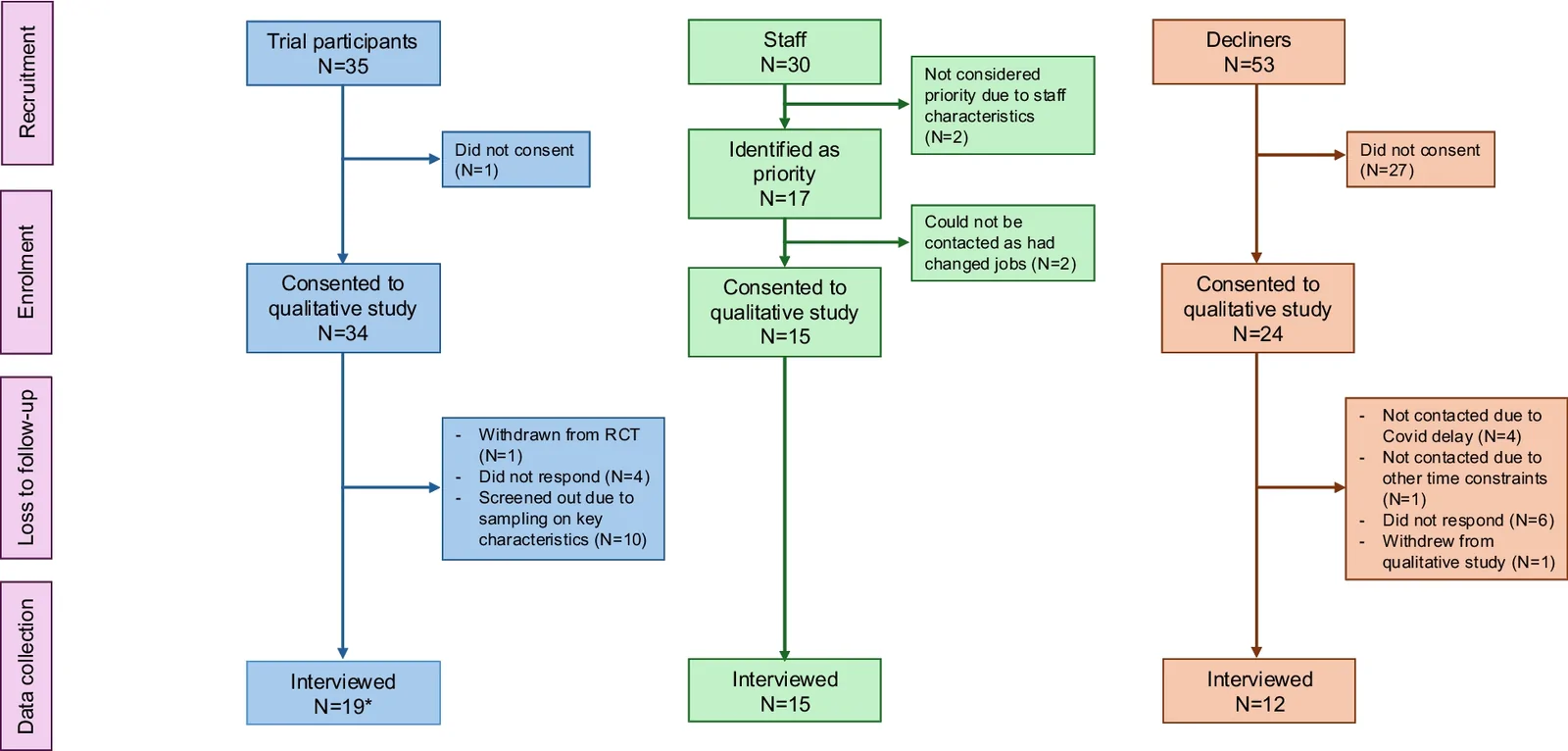
Flow chart of participant recruitment *Included one email interview

All but one (*n = *34) trial participants consented to be contacted for interview (Fig. 1). To ensure balance in sampling characteristics, after completing 14 interviews we reviewed participant characteristics and thereafter prioritised interviewing participants with underrepresented characteristics. Eighteen interviews were conducted by telephone (11 participants allocated to intervention, and seven to usual care) with one interview by email, at the participant’s request. Interviews were completed between May 2020 and August 2022, and the mean duration of telephone interviews was 22 min (range: 10–64 min).

Twenty-four decliners consented to the qualitative study, of which 12 were interviewed from four study sites. Interviews completed between May 2020 and October 2022. The mean interview duration for decliner interviews was 12 min (range: 5–22 min).

For staff interviews, we screened all 30 eligible staff and identified 17 priority interviews based on capturing a range of staff characteristics. Fifteen staff interviews were completed across all seven study sites. Staff interviews took place between July 2022 and November 2022. The mean interview duration was 29 min (range: 16–51 min).

In total, we conducted 46 interviews, with our recruitment target met for all three participant groups. Full details of participant recruitment are presented in Fig. 1. We achieved a maximum variation sample for key characteristics for the trial participants (Table 3), and staff interviews (Table 4) as planned, within the trial constraints.

**Table 3:** Sampling characteristics for trial participants (*N = *19)

Characteristic		N	(%)
Treatment allocation	Intervention	11	(57.9)
	Usual care	8	(42.1)
Trial status	Completer^b^	16	(84.2)
	Dropout^c^	3	(15.8)
Study site^a^	1	6	(31.6)
	2	2	(10.5)
	3	5	(26.3)
	4	0	(0)
	5	5	(26.3)
	6	1	(5.3)
	7	0	(0)
Pregnancy number	0	10	(52.6)
	> = 1	9	(47.4)
Hospital admission in the year prior to pregnancy	Yes	6	(31.6)
	No	13	(68.4)
Crises in the year prior to pregnancy	Yes	11	(57.9)
	No	8	(42.1)
Genotype	HbSS^d^	10	(52.6)
	HbSC^e^	8	(42.1)
	Other	1	(5.3)
Self-reported transfusion history at RCT enrolment	None	9	(47.4)
	Top-up only	4	(21.1)
	Exchange only	1	(5.3)
	Top-up and exchange	4	(21.1)
	Not known	1	(5.3)

^a^Participants recruited to RCT by study site:1 (*n = *11), 2 (*n = *1), 3 (*n = *7), 4 (*n = *7), 5 (*n = *7), 6 (*n = *2), 7 (*n = *0)

^b^Included one participant who had first SPEBT and was withdrawn from further SPEBT following a transfusion reaction

^c^Dropped out of intervention, though included in quantitative intention-to-treat analysis

^d^HbSS = homozygous sickle cell

^e^HBSc = Haemoglobin Sickle C disease

**Table 4:** Sampling characteristics for staff (*N = *15)

Characteristic		N
Staff role	Consultant obstetrician	3
	Consultant haematologist	4
	Research Midwife	5
	Research Nurse	1
	Clinical Research Practitioner	2
Study site	1	3
	2	2
	3	2
	4	2
	5	2
	6	2
	7	2

### Findings

The finding reports the combined data from the interviews with 19 trial participants, 12 decliners and 15 staff participants.

We identified three overarching themes: (1) Factors affecting patient decisions on taking part in the trial; (2) experiences of the TAPS2 trial; (3) recommendation for a future RCT. Each theme incorporated several subthemes summarised in Table 5. Themes 1 and 3 incorporated data from all three participant groups, enabling triangulation of the whole data corpus, while Theme 2 reflects the findings from the trial participant and staff data only. Illustrative verbatim quotations from the interviews are reported in the text.

**Table 5:** Key findings

Key findings		Trial participants	Decliners	Staff
Theme 1. Factors affecting patient decision-making on trial participation				
Positive motivations for trial participation	Hope of benefit to their own health	✓	Decliners wanted to help others with SCD but not to receive transfusions	Staff reported hearing these motivations from patients
	Benefit to the pregnancy and their baby’s health	✓	*As above*	*As above*
	Wish to support research for the benefit for others with SCD	✓	*As above*	*As above*
Concerns about SPEBT	Risk of infection	✓	✓	Staff reported that patients expressed these concerns about transfusions
	Risk of being given the wrong blood	✓	✓	*As above*
	Other adverse reactions	✓	✓	*As above*
	Concern that SPEBT could trigger sickle crises	✓	✓	*As above*
	Fear of SPEBT causing problems in current pregnancy if not needed in previous pregnancy	✓	✓	*As above*
	Concerns about cannulation	✓	✓	*As above*
	Cultural stigma around receiving blood	X	X	Cultural stigma was mentioned by one clinician but not by any patients
Perceptions of the benefit or relevance of SPEBT to participants	Benefits to mother	✓	Decliners felt that either their SCD was too mild to need SPEBT, or it was not worth the risks	✓
	Benefit to baby	✓	*As above*	✓
	Reduction in sickle crises	✓	*As above*	✓
* Weighing up risk vs benefits of SCD*	Severity of SCD affects perceptions of risk vs benefits	✓	*As above*	✓
	Women with milder SCD less likely to view SPEBT as relevant or beneficial to themselves	✓	*As above*	✓
Partner or family influence on perceptions of transfusions	Influence of family in patient’s decision to take part (or not)	✓	✓	✓
	Role of partner in decision	✓	✓	✓
	Importance of a good patient-clinician relationship	✓	✓	✓
	Some women of colour might feel less trusting of a white healthcare professional	X	X	Reported by one clinician
Trial Burden	Time, cost and effort of travel, arranging childcare or time off work	✓	✓	✓
	Participation could place an additional burden on women already coping with pressures of SCD, work and/or caring for other children	✓	✓	✓
Theme 2. Experiences of trial				
Recruitment process: patient’s experiences	No significant concerns were highlighted by patients regarding their experience of the recruitment process	✓	✓	These findings concurred with staff reports of feedback from patients to them
	Recruitment materials appeared generally acceptable to patients, although one participant suggested including more pictures and visual aids to improve readability	✓	✓	*As above*
Recruitment process:staff experiences	Recruiting staff reported challenges in identifying eligible women and completing all entry criteria early enough in pregnancy to enrol the patient into the trial before six weeks gestation (the recruitment cut off point)	N/R	N/R	✓
	At some sites with linguistically diverse populations, need for translation had presented difficulties	N/R	N/R	✓
Outcome measures – patient reported outcome measures	Questionnaires appeared generally acceptable to all trial participants	✓	N/R	Research staff confirmed general acceptability of questionnaires to patients
	Trial participants suggested including additional outcomes focussing on impact of SCD on employment, and mothers’ perceptions of how pregnancy was going	✓	*As above*	*As above*
Control group experiences	Trial participation was acceptable and not burdensome	✓	N/R	✓
Participants positiveexperiences of SPEBT during the trial	*.* Benefits included more energy, less pain, reduction or stopping of crisis for some women, fewer hospitalisations, a sense of ‘feeling better in myself’ or an overall sense the current pregnancy was going better than previous pregnancies	✓	N/R	✓
Participants negative experiences of SPEBT	Side effects (dizziness, pins and needles, headaches and one report of citrate toxicity)	✓	N/R	✓
	Issues with cannulation	✓	N/R	✓
	Effort/cost of attending appointments	✓	N/R	✓
Reasons for patients withdrawing from trial	All three patients who dropped out of the intervention had hoped to be allocated to usual care but had been randomised to SPEBT	✓	N/R	Reasons for withdrawals not mentioned in staff interviews
	One began transfusions but withdrew from the intervention after difficulties in cannulation	✓	N/R	*As above*
Theme 3. Improving recruitment and trial experiences for future trials				
Improving trial recruitment- recommendations	Raising awareness of:		N/R	
	▪ the effects of pregnancy on SCD	✓		✓
	▪ potential benefits of SPEBT	✓		✓
	▪ the TAPS2 trial findings	✓		✓
	Raising awareness through social media, internet websites and posters in hospitals	✓	N/R	✓
	Recruitment information needs to be:			
	▪ easy to understand, e.g. using visuals/videos to increase accessibility	✓	✓	✓
	▪ accessible to partners & family	✓	X	✓
	▪ available earlier in pregnancy if possible	✓	X	✓
	Value of video peer testimonies	✓	N/R	✓
	Staff recommended raising wider awareness among clinical colleagues through existing clinical networks	N/R	N/R	✓
Improving trial experiences and conduct – key recommendations	Support with childcare and travel costs to attend trial appointments	✓	N/R	✓
	Providing pre-transfusion advice to patients on how to reduce issues with venous access	✓	N/R	N/R
	Adding outcome measures on impact on employment, and on how the mother herself felt that the pregnancy was going	✓	N/R	X
	Ensuring adequate apheresis capacity, for example through additional funding or ringfencing resources	N/R	N/R	✓
Future study design	Clear support from all interviewees to include an observational study arm to the design of a future trial for women who do not want to be randomised, or ineligible for the trial	✓	✓	✓

√ indicates finding supported by data from this interviewee group

X indicates that issue was not mentioned by this interviewee group

*N/R* Question/s were not relevant to participant group, so no response is recorded

### Theme 1. Factors affecting patient decision-making on trial participation

Patients described five factors informing their ultimate decision. Whilst all participants generally reported multiple reasons, decliner’s ultimate decision (i.e. to not participate) was always driven by their reluctance to receive the intervention, which overshadowed any other factors.

#### Positive motivations for trial participation

Health benefits were a key motivating factor. Trial participants hoped that participating could benefit their own health, their pregnancy and/or their baby’s health. They felt the trial could provide health benefits both directly, from potentially receiving transfusions and indirectly, from the extra clinical attention and monitoring involved.

*I just wanted to do what was best, just to help my baby and myself* (Trial participant 3, intervention arm, completer*)*

Altruistic motivations were commonly reported by all patients, including decliners; they wanted to support research to benefit their SCD community, rather than solely for personal health gains. Staff also concurred altruism was a key motivating factor. Both patients and staff perceived SCD as an under-researched and neglected condition. Participating was therefore also seen as a way to raise awareness of SCD as well as potentially finding better treatments for SCD during pregnancy. Whilst most trial participants reported altruism as a contributing factor alongside their hope for health benefits, one trial participant’s decision was primarily altruistic; she had no expectations her own health would improve from being in the trial.

*“I did not see how I was going to benefit personally from it but... I thought if it was going to help people out then I will do it.”* (Trial participant 12, intervention arm, completer)

In contrast, whilst the decliners wanted to support research for their community, their concerns about SPEBT meant they felt unable to join this trial.

#### Views on blood transfusions

Patients’ views on transfusions and treatment preferences emerged as the key determinant in their decision about joining the trial. Decliners primarily refused because they did not want SPEBT; all but one of the decliners, wanted to support this research and would have participated if they could have been allocated to the usual care arm:

*“If I had the option…where I wouldn’t have had the transfusion, I would have been involved.”* (Decliner, participant 7)

Among the trial participants interviewed, four cited a preference for SPEBT, four preferred usual care, and the rest had no clear preference. Three of the four who preferred SPEBT had severe disease and hoped SPEBT would reduce their risk of crises during their pregnancy:

*“I was hoping…the computer would have chosen me to have the [blood] exchanges… I couldn’t imagine going through pregnancy with more crises...”* (Participant 2, intervention arm, completer)

Participants with no preference—regardless of disease severity—were either unable to make their mind up or else felt both treatment arms had advantages for them. This latter group were confident they would receive any care they needed, regardless of which arm they were randomised to.

Participants citing a preference for usual care had less severe SCD disease. They wanted usual care either because they wanted to avoid transfusions or felt being in the usual care would be ‘slightly less onerous” in terms of the trial burden. Staff also identified reluctance to receive transfusions as the main recruitment barrier. This highlights the importance of patients’ transfusion related concerns, which is supported by the triangulation of evidence from all participant groups. Concerns regarding the perceived risk of potential harms from SPEBT were common among those hesitant about transfusions.

#### Concerns about SPEBT

Patients described a range of concerns about SPEBT, mainly focused on the risks associated with transfusions. These included fears about infection from blood or cannulation, receiving the wrong blood, and adverse reactions:

*“I was more worried… about being given the wrong blood… and infected blood.”* (Trial participant 9, intervention arm, completer)

For some decliners, SPEBT itself was also seen as potentially harmful. One woman who experiences a few crises each year, was worried that exchanging blood could trigger a sickle crisis, rather than prevent one:

*“Take old blood out and put new blood… maybe that is going to cause some crises.”* (Decliner, participant 10)

Staff consistently reported that patients concern about blood safety were common. Several clinicians observed these concerns were particularly among women with cultural backgrounds and/or experiences from living in regions with high HIV prevalence. These women understandably required additional reassurance about the screening processes and infection risk:

*“It’s like, is it safe? Has the blood been screened? Could you get an infection from HIV?... people with sickle cell from an Afro Caribbean background are needing that reassurance.”* (Staff interview 8, Research Midwife)

Others feared that starting a new intervention might negatively affect their pregnancy. Women with previous losses or IVF pregnancies felt that introducing any new intervention could jeopardise an already fragile pregnancy and would lead to regret. For this decliner, pregnant via IVF with a history of recurrent miscarriage, avoiding SPEBT was perceived as a safer choice.

*“Everything is going well, I don’t want regrets… or to do anything that might affect it.”* (Decliner, participant 1)

In contrast, women with previous successful pregnancies without the need for transfusion, also questioned why they would now need one, fearing transfusions might cause problems and a less successful pregnancy outcome:

*“[They’d] had children before and not needed one, so they couldn’t understand why it was a good idea to do it.”* (Staff interview 1, Research Midwife)

Prior experience of transfusions — or lack of experience — were also important. Patients who had never had a transfusion before, described feeling uncertain or frightened by the idea:

*“It sounds scary, like they’re going to take out all the blood I have and give me new one.”* (Decliner, participant 1)

Concerns were also not limited to the transfusion with several trial participants also voicing apprehensions about cannulation too. For example, they were worried that the cannulas could ‘injure’ their veins; those participants, who knew they would require a femoral line, were particularly concerned about this.

*“I was just a bit worried about the cannulation… it’s going in my [groin] like.”* (Trial participant 19, intervention arm, completer)

Clinicians acknowledged that patients’ transfusion related concern was a complex issue, reflecting the interplay between patients actual, current clinical SCD risks, and myths or misapprehensions (e.g. based on previous times when blood safety procedures were less rigorous). One clinician, a haematologist, also reflected on the paradoxical nature of recruiting patients for regular SPEBT as part of a trial, when they routinely advised patients to avoid unnecessary transfusions.

*“We spend a lot of time saying ‘don’t let doctors transfuse you if you don’t need it,’ and then we’re saying, ‘we want you to have a transfusion as part of this trial.”* (Staff interview 6, Consultant Haematologist)

All clinicians recognised the complexities their patients faced in balancing the risks and benefits of SPEBT and their role in supporting patients to make the best decision for their baby and themselves. For patients trying to weigh up the risks vs benefits of SPEBT for their pregnancy, possible benefits from SPEBT presented the other side of the risk–benefit equation.

#### Perceptions of the benefit or relevance of SPEBT

Perceptions of benefit from SPEBT were linked to patients views of their SCD severity. Those with a history of frequent and painful sickle crises wanted SPEBT to reduce the number of crises during their pregnancy. In contrast, those with who reported milder SCD, saw no potential benefits from SPEBT, as they were well without transfusions. These included first time mothers, as well as those who had previously successful pregnancies without transfusions.

*“I have 2 kids and did not need any transfusions, and I did not want to risk it this time.” *(Decliner, participant 3)

Some decliners, with milder SCD, also expressed ethical concerns about receiving SPEBT, viewing blood transfusions as a scarce resource that should be reserved for patients with greater clinical need. Staff interviewees also reflected on the difficulties of trying to recruit this group of patients with mild SCD who did not feel they needed any intervention.

#### Partner or family influence on perceptions of transfusions

Partners and wider family networks (including parents, siblings, aunts, and grandparents) often played a major role in shaping patients’ views about transfusions and their decision about joining the trial.

*“I had conversations with my husband, my dad, my mum, my sister and… we all had a family conversation about [the trial] and the risks.”* (Trial participant 2, intervention arm, completer)

Staff also reported that family opinions could sway patients either towards or away from participation. Those involved in recruitment, especially nurses and midwives, described cases where relatives strongly discouraged transfusion, as well as situations where family members supported trial involvement:

*“She came with an aunt who said no… ‘nobody gets blood [in the family] and they are fine’; and equally I had someone else came with a family member who said… ‘this is for your good… listen to the doctor.’”* (Staff interview 13, Consultant Obstetrician)

Clinicians highlighted the importance of partner role in patient’s decision making, but it could be challenging to address their concerns, when they were not at the appointment and information was relayed second-hand via the patient. As one haematologist described, family concerns were especially understandable where there was a known case of fatal complications following transfusion, leading to fears that this could happen again:

*“There are people in the community who have died because of blood transfusions… that is in the psyche of the patient population.” *(Staff interview 6, Consultant Haematologist)

Staff also discussed the importance of a good patient–clinician relationship to enable patients to fully discuss their feelings and concerns. It was important to take time during recruitment to provide reassurance and information, and empower patients to make an informed decision. Building rapport and trust was essential to reassure patients that their clinician genuinely wanted the best for them and their baby, rather than simply wanting to recruit them into the trial.

*“You have to get a rapport… so they trust you. You don't want them to feel like you are using them just for research.”* (Staff interview 8, Research Midwife)

Several clinicians noted that past negative healthcare experiences could lead to patient’s mistrusting health care professionals, particularly amongst those felt their symptoms had previously been dismissed. Race was also mentioned, by staff only, as another potential barrier in eliciting trust; one white midwife reflected that some women of colour might feel less comfortable with a white recruiter, although this was not openly expressed by any patients.

*“I do wonder whether the colour of my skin was an issue* *because I don't have black or brown skin* *… I just wonder whether that might have been a barrier, but I never asked.”* (Staff interview 7, Research Midwife)

#### Trial burden

Participants, decliners, and staff all highlighted the practical burden of trial participation, particularly the time, cost, and effort of travelling to extra appointments, often requiring childcare or time off work.

*“I would have to come to the hospital more often… I would need to look for childcare for the whole day.”* (Decliner, interview 2)

Clinicians noted that these demands added pressure for women already managing the physical and emotional challenges of SCD alongside their work and family responsibilities:

*“It's quite a difficult population to sell another treatment to as they have to endure as much as they do.”* (Staff interview 1, Research Midwife)

Despite these challenges, the TAPS2 trial did successfully recruit.

Finally, for contextual background, trial recruitment took place during and after the emergency phase of the COVID‑19 pandemic, when participants in this high‑risk group were shielding until vaccinations become available, from April 2021 onwards. Nevertheless, COVID‑19–related health concerns did not appear to play a major role in influencing decisions about trial participation. Only one decliner cited the pandemic as a contributing factor. A few trial participants reported some concerns about potential exposure, but most did not consider this impacted their decision-making; however, one trial participant explained that being on furlough made participation easier.

### Theme 2. Experiences of trial

#### Recruitment process

Trial participants reported no major issues with the recruitment process. Recruitment materials appeared generally acceptable to patients; the only recommendation, from one participant was to include more pictures and visual aids to improve readability.

In contrast, recruiting staff experienced several challenges. These included identifying eligible women early enough in pregnancy before the recruitment cut off at 18 weeks. This was particularly problematic when additional patient notes were required for ‘out of area’ patients.

Pandemic‑related disruptions also impacted recruitment, especially during the emergency phase. Challenges included: inability to recruit when trial activity was paused for 6 months; reduced staffing as staff were redeployed; delays in booking appointments; challenges communicating the complex trial information remotely; and limited clinical capacity when services resumed.

“*We had a big gap in recruitment… many of the nurses were still redeployed*” (Staff interview 9, Research Midwife).

Additionally, language barriers presented an additional challenge at hospitals sites to recruit non-English speaking patients. Staff were cautious about asking family members to translate, as this could influence patients’ decisions, while providing professional interpreters was not feasible, especially at hospitals serving multilingual communities where many languages were involved.

*“You've got 200 languages spoken… it would take a lot of resource*.” (Staff interview 1, Research Midwife)

#### Study conduct

All participants found the questionnaires easy to complete. A few suggested additional items for future trials (discussed in Theme 3). For control arm participants, questionnaires were the only study procedure, and none found participation burdensome. This contrasted with the intervention group, who reported the added time, cost, and logistics of attending transfusion appointments.

#### Participants experience of SPEBT during the trial

Interviews with the trial participants and staff revealed both positive and negative experience from SPEBT. Participants in the intervention arm reported multiple benefits, including more energy, fewer crises, less pain, fewer hospitalisations, and overall improved wellbeing.

*“ I didn't have any crises… so we saw it was working.”* (Trial participant 7, intervention arm, completer)

Several participants said their partners also noticed improvement in their energy levels following the SPEBT:

*“My husband noticed I was more tired before the next one was due… after the transfusion I would perk up again.”* (Trial participant 20, intervention arm, completer)

Clinicians also observed improved health outcomes following the transfusions, and particularly so in those patients with more severe disease, requiring frequent admissions:

*“Compared to previous pregnancies… they've been that much better and flown through the pregnancy… not had crises* *and did not need any emergency transfusions.”*

(Staff interview 12, Consultant Haematologist)

Negative experiences related to treatment side effects, cannulation and logistical challenges to attend the transfusion sessions. Most side effects were within the range of common, anticipated side effects from SPEBT including dizziness, pins and needles and headaches. However, a few participants experienced more common but severe side effects including occasional citrate toxicity. One participant who explained she had a severe adverse reaction requiring hospital admission, resulting her treating clinician withdrawing her from further SPEBT transfusions.

*“I had a reaction… a big one… I had to be admitted [to hospital]”* (Trial participant 5, intervention arm, completer but intervention discontinued)

Cannulation was also challenging for some participants. While the two women who required a femoral line described the procedure as “unpleasant”, it was those cannulated in the hand or arm who reported pain, bruising, and for some, frustration when nurses experienced difficulties obtaining venous access. Negative cannulation experiences posed a potential barrier to retention: one participant seriously considered withdrawing from the trial because of painful cannulation (although she ultimately completed the intervention), whereas another withdrew due to the pain and distress associated with repeated cannulation attempts.

*“It was very painful and sore so I literally cried I can't tolerate this anymore... they used the guidance cannulation [which] also failed for three to four times then I was like, now it's enough.”* (Trial participant 15, intervention arm, dropped out from the intervention)

Finally, many women described the significant practical effort required to attend the transfusion sessions. These logistical challenges included arranging childcare and/or taking time off work as well as travel with its associated costs:

*“It was juggling a two-year-old, working, being pregnant… I had to pay for childcare and transport to London.”* (Trial participant 2, intervention arm, completer)

#### Reasons for patients not receiving intervention as intended

Of those interviewed, three trial participants declined SPEBT after being allocated to the intervention arm, as they had hoped to receive usual care, highlighting the importance of treatment preferences within a RCT study design. Additionally, as described earlier, another participant discontinued the intervention following an antibody reaction, whilst another declined further attempts at SPEBT following an initial unsuccessful, and painful, cannulation attempt.

### Theme 3. Improving recruitment and trial experiences for future trials

All participants groups were asked for suggestions to inform the future definitive trial of SPEBT. The findings identified three subthemes: recommendations on improving recruitment; recommendations on improving the experience and/or delivery of the trial itself; and the study design.

#### Improving trial recruitment

Participants, decliners, and staff suggested raising awareness of both SPEBT and the clinical trial through social media, NHS and charity websites, and posters in participating Trusts. This could help women learn about the trial before being approached by clinicians:

*“…it can be quite intimidating… when they’re approached by a health care professional… giving them some information on social media… posters… TikTok… just to build awareness… so many times I approached women and they had no idea we did research…”* (Staff interview 7, Research Midwife)

Staff also recommended increasing awareness among clinical colleagues through existing clinical networks such as regional Haemoglobinopathy Coordinating Centres (HCCs).

Across interviews, staff and many participants emphasised the importance of clear, accessible information to support patients’ decision making when weighing up the risks and benefits. Clinicians considered patient education was vital from observing how patient’s concerns could be exacerbated by out-dated information or myths about transfusion safety. Together, participants recommended information covering pregnancy related SCD risks; the SPEBT procedure; blood safety and side effects; as well as the potential benefits for mother and baby related SCD risks. Video peer testimonies from patients own experiences of transfusion during their pregnancy were seen as particularly powerful and trusteed approach for patients.

*“Someone with sickle cell talking [in a video] is more powerful than someone like me… from an authoritarian side of things.” *(Staff interview 1, Research Midwife)

Making information available online could also help partners and families, who often influence decisions. Participants also highlighted the value of lay language, pictures, and videos for multilingual audiences:

*“Sometimes when there’s a lot of information on paper, it can be overwhelming… having it broken down visually can be helpful.”* (Decliner, participant 11)

Several staff also suggested offering online information sessions combining information likely of topical interest, alongside information about the trial.

#### Improving trial experiences

Key recommendations from participants included clearer pre-transfusion advice, especially about hydration to ease cannulation:

*“…make sure… you have a good breakfast, drink lots of fluids… that will really help… with your blood vessels.”* (Trial participant 2, intervention arm, completer)

Participants juggling childcare and long journeys suggested providing support with travel and childcare costs; staff also agreed travel expenses would help mitigate trial burden and allow more women to participate. Most participants were satisfied with the proposed trial outcome measures, though some suggested additional patient reported outcomes such as the impact of SCD on the patient’s occupation (employment or homecare tasks), experiences of transfusion reactions, and perceptions of their pregnancy progress.

The main recommendation from staff, was the need to ensure sufficient apheresis capacity in any future trial, for example through additional funding or ringfencing resources. Whilst provision of SPEBT for trial patients had not been an issue at some sites, at other sites it had felt a major struggle.

*“We’ve really struggled in terms of staffing… no sickle midwives… small apheresis team… every exchange we had to beg for people… a future study would need much better funding.” *(Staff interview 11, Obstetric Consultant)

#### Future study design

After interviews began, participants and decliners were asked whether they would take part in a future observational study arm within the definitive RCT. There was strong support for this from both trial and decliner participants, with 11 of 12 decliners stating they would have participated if an observational study arm existed.

*“I would have happily taken part… questionnaires and focus groups I would happily be part of, but anything intrusive I wouldn’t do.”* (Decliner, participant 9)

Staff also unanimously supported the inclusion of an observational arm to the trial, explaining this approach would capture outcome data which would otherwise be missed i.e. from patients unwilling to be randomised, as well as those patients already receiving SPEBT, who would otherwise be excluded from trial participation.

## Discussion

This qualitative study explored the views of trial participants, decliners and staff on the feasibility and acceptability of a RCT comparing SPEBT versus usual care in pregnant women with SCD. Whilst anecdotal evidence suggests SPEBT is increasingly used for this group in clinical practice, limited evidence means it is not yet included in clinical guidelines.

### Key findings and interpretation in the context of existing research

This is the first qualitative account of patient and staff experiences within a feasibility RCT of SPEBT during pregnancy and was designed to inform the design of a definitive trial. The women eligible for TAPS2 occupy intersecting identities – as pregnant women, as people living with a serious chronic, condition; and, as SCD in the UK predominantly affects people of Black African and Black Caribbean heritage, members of minority ethnic groups [2]. This influences both their decisions about participation and their trial experiences.

While prior research has explored trial participation among people with SCD [32, 33], this is the first to specifically focus on pregnant women within the context of a transfusion-based RCT. Across all participant groups, the dominant factor influencing (or perceived to influence) willingness to participate centred on weighing the perceived benefits of SPEBT against its risks, particularly in terms of evaluating the risks in relation to fetal wellbeing. This reflects evidence that fetal benefit is the primary motivator for participation in pregnancy research [34–38], with maternal benefit and altruism secondary [38]. Both decliners and trial participants recognised the importance of research, and the fact that many decliners still joined the qualitative sub‑study stemmed from concerns about SPEBT itself rather than research participation.

In the specific context of SCD, previous mixed-methods and qualitative studies describe how patients draw on lived experience when evaluating risks and benefits [32, 39]. Our findings mirror these: participants made careful, deliberative decisions informed by past disease experiences, symptom severity, and expectations of transfusion. Effective clinician communication shaped their decisions, consistent with previous research highlighting the importance of supportive clinician rapport for shared decision-making [40, 41].

Hesitation commonly stemmed from concerns about transfusion safety—including infection, adverse reactions, or triggering a sickle crisis—concerns consistent with research on intervention acceptability [42]. For women with milder SCD or prior uncomplicated pregnancies, concerns were amplified by uncertainty about whether SPEBT was clinically necessary; reflecting previous literature highlighting positive attitudes and beliefs in treatment facilitates recruitment [43]. Echoing community SCD research, trusted and culturally appropriate information was essential [33]. Broader survey findings similarly highlight the influence of daily-life burden, clarity of information, and perceived risks on decision-making [44].

Partner and family influences were also important. Negative transfusion experiences among relatives or community members amplified caution. Unlike findings from some pregnancy trials where partner views have limited impact [36], family perspectives appeared more influential in this study—potentially reflecting both the higher-risk nature of the intervention and the concentration of SCD within families and close-knit communities.

Recruitment was affected by COVID-19, including delayed referrals, service pressures, and conflicting public health messaging jeopardised enrolment before the 18-week cutoff. Similar challenges have been reported in a mixed-methods study examining the experiences of people with SCD during the pandemic [45]. Staff also reported difficulties accessing apheresis capacity, reflecting wider pressures in UK transfusion services [46].

Participants generally reported positive trial experiences. Those receiving SPEBT noted improved energy, fewer crises, and reduced hospitalisations. One participant had a severe autoimmune reaction to SPEBT who remained in the trial but was withdrawn from further trial transfusions: no unexpected side effects were reported among other intervention participants. Another intervention participant also dropped due to repeated unsuccessful cannulation attempts. Practical burdens—travel, childcare, time—were common but consistent with broader research on trial participation in pregnancy [42, 47]. Questionnaire burden was minimal as data collection was embedded in routine care.

Finally, these findings must be viewed within broader sociocultural contexts. SCD is widely recognised as a ‘racialised’ condition [48, 49] associated with stigma and bias [45, 50, 51], and inequitable care [48, 49, 51]. Many individuals with SCD experience a lack of timely and appropriate care [52]. Patients’ expert knowledge of their own illness [53] often contrasts with clinicians’ limited understanding, contributing to mistrust. Whilst US research has identified distrust of the medical community as a barrier to participation in clinical trials [54], the evidence base for the UK is less clear [55]. Only staff explicitly raised this as a potential barrier; however, patient participants may have been unwilling to disclose these sensitive concerns during interviews. This underscores the need for culturally sensitive, trust-building approaches to recruitment and consent, consistent with recommendations from community-engaged SCD research [33] and ethical arguments supporting the inclusion of pregnant women in research [56].

### Recommendations for future definitive RCT of SPEBT

A future trial should incorporate recruitment strategies addressing concerns across diverse groups of pregnant women with SCD—those with mild or severe disease, those with positive or strained relationships with clinicians, and those with negative prior medical experiences. Approaches should consider how women evaluate the risk/benefit of SPEBT. We propose three complementary strategies to support future studies on this topic.

#### Patient education

Enhanced education may reduce recruitment particularly for women with ‘mild’ SCD, those with previously uncomplicated pregnancies, limited awareness of increased pregnancy related risks, no prior transfusions, or heightened anxiety about treatment risks. Recommendations on patient education are summarised in Table 6.

**Table 6:** Recommendations on patient education strategies to address barriers to recruitment and support patient decision-making

Content of patient education
• Information needs to be balanced and focussed on issues of most concern to patients, as well as addressing common myths or misunderstandings about transfusions
• Participants recommended the use of videoed peer testimonies from patients who have received SPEBT during pregnancy to explain the treatment procedure, their experiences and benefits and side effects, which will also allay cultural mistrust
• Information needs to include the increased risk of SCD complications during pregnancy, as well as the potential benefits of transfusion, along with information about the safety and risks of blood transfusions from trusted sources
• Inclusion of videos and information from trusted clinicians, to address those patients with mild SCD who are concerned about the risks and see no benefit from receiving transfusion. Some staff recommended inviting women to come and visit the day unit and talk to other women having transfusions
• To make patients aware of the trial, earlier in pregnancy or ideally even before they became pregnant through more posters in hospital clinics, online information including websites and social media, patient support organisations
Delivery of patient education
• Online information and social media offer a means of getting information to patients not yet recruited, as well as to partners and families who may contribute to the patient’s decision
• Information needs to be inclusive to recruit from a diverse population e.g. translated or use of pictures/videos to ensure potential participants with limited English or their partners or families are not excluded
• To make patients aware of the trial, earlier in pregnancy or ideally even before they became pregnant through posters in hospital clinics and online information including websites and social media, patient support organisations

#### Study design

Adapting the study design by including an observational arm or a patient preference design could accommodate those with a strong treatment preference, as well as those currently excluded from the trial such as those already receiving SPEBT. Women with SCD in pregnancy are a small patient population and assessing clinical outcomes for across the full cohort is crucial. Many decliners indicated they would have taken part in an observational study, and some randomised participants withdrew due to not wanting SPEBT.

#### Additional support for specific patients

In addition, additional support is recommended for patients who mistrust the intervention and/or clinicians – especially those referred into specialist centres or with relevant negative prior experiences. Clinicians should build rapport and trust so patients feel safe to share their concerns.

To reduce the risk of non-adherence which could bias trial outcomes, patient treatment preferences should be explored, and randomisation explained clearly. Finally, there is a need to provide financial or practical support to reduce participation costs, especially for those receiving the intervention, to ensure inclusive recruitment.

### Strengths and limitations

Our study achieved the planned sample size and through purposive sampling, captured a wide range of experiences and perspectives. We generated coherent findings that deepen understanding of barriers to participation in this study population. A key limitation to this study was the brevity of some of the interviews—particularly those with decliners – consistent with literature demonstrating shorter interviews with decliners due to the narrower scope of topics and less investment in the research [57, 58]. Several trial participant and staff interviews were abridged due to immediate clinical or personal demands (such as the need to respond to a newborn). Although some interviews were briefer than expected, they still provided sufficient content (as demonstrated in Table 5) to identify consistent themes across participant groups, and to inform recommendations.

Many staff interviewees reflected on the issue of patient-clinician trust and its impact on study recruitment and participation, though this issue was not raised by trial participants or decliners. It is important to acknowledge that this – and indeed the other perceived barriers that were voiced by staff – may not have been significant concerns for women themselves, but instead reflect assumptions or interpretations made by staff on their behalf. Additionally, conducting telephone interviews with White researchers may have made it harder for women of colour to discuss sensitive concerns.

Interviews were conducted during the COVID-19 pandemic, when individuals living with SCD were designated as “extremely clinically vulnerable” and advised to shield at home. This was also a period of heightened concern about health and risk. Although our topic guides included questions about the pandemic, it is unclear to what extent the broader context may have more subtly shaped experiences of study participation and recruitment.

This study focused on a small, specific population—pregnant women living with SCD in England—with the overall aim of informing a future definitive RCT. Despite this narrow focus, the research generated insights that may have wider generalisability. It highlighted the particularly pronounced importance of family perspectives in patient decision-making within transfusion-related research during pregnancy. It also emphasised the need to consider incorporating observational arms within future trials to promote equity of access for patients who are unable or unwilling to participate in a RCT.

The novelty of these findings is reinforced by the fact that only in the last few decades have women with SCD had realistic expectations of motherhood, resulting in limited research in this area. Individuals with SCD represent a unique population navigating multiple intersecting identities. Living with a rare chronic condition often means they are highly knowledgeable about their health—sometimes more so than members of their treating clinical team—and adept at managing frequent interactions with healthcare services. These findings may therefore have relevance beyond SCD, particularly for other patient groups living with chronic disease who are considering or experiencing pregnancy, where decision-making is shaped by complex medical, social, and familial factors.

## Conclusion

Recognising and addressing the unique concerns and treatment preferences of pregnant women living with SCD through empathetic, participant-centred recruitment and trial design is essential for maximising recruitment in future trials. Women’s treatment preferences and evaluation of the risk-benefits of the intervention, played a key role in their decision to take part in this feasibility trial. These factors need to be considered to aid optimal recruitment and avoid unnecessary ‘non-adherence’ which could potentially bias a future trial. Incorporating an observational study arm as part of a future definitive RCT, along with other strategies, would strengthen the evidence base to improve understanding of outcomes for pregnant women with SCD.

## Supplementary Information

Additional file 1. Final topic guide for trial participants.

Additional file 2. Final topic guide for trial participants.

Additional file 3. Final topic guide for staff participants.

## Data Availability

The datasets used and/or analysed during the current study are available from the corresponding author on reasonable request.

## Abbreviations

RCT: Randomised control trial
SCD: Sickle cell disease
SPEBT: Serial prophylactic exchange blood transfusion
TAPS2: The Transfusion Antenatally in Pregnant Women with Sickle Cell Disease study
Completers: Patients who joined the TAPS2 trial and completed participation according to protocol
Dropouts: Patients who joined the TAPS2 trial and withdrew from the intervention
Decliners: Patients who were invited into the TAPS2 trial but declined to participate
