# Supplementary material for: The acceptability and feasibility of a randomised controlled trial of serial prophylactic exchange blood transfusion versus usual care in pregnant women with sickle cell disease: a qualitative study
Source: BMC Pregnancy Childbirth. 2026 Jul 3;26:1070. doi: 10.1186/s12884-026-09244-5 (PMC13617929; doi:10.1186/s12884-026-09244-5)
Supplement: Supplementary file 1 [file 12884_2026_9244_MOESM1_ESM.docx]

**INTERVIEW QUESTIONS <audio recording commences>**

Before we begin, I know you have already provided written informed consent to take part in this interview, I would like to double check you still agree to take part in this interview and are happy for me to record this interview. Please could you confirm this for the recording?

**QUESTIONS FOR ALL PARTICIPANTS**

1. Please could you briefly tell me why you took part in this trial?
2. Have you taken part in any research before? Did this influence your decision about this study at all

Was this your first pregnancy or have you had other pregnancies before? When were your other pregnancies? How were your previous pregnancies?

3. Could you tell me a little about how you have been with your sickle cell in the few years before your pregnancy? *(Prompt: Have you had any crises? Hospital admissions?)*
4. Before this pregnancy, had you ever had a transfusion? *(Prompt: could you tell me what type of transfusion you had, and why?) (If had previous pregnancies could specifically ask if every had a transfusion when pregnant before the current pregnancy)*
5. This was a randomized trial which means that people taking part are assigned by chance, rather than by choice, to either the intervention group or the control group.
   a) What are your views on being randomized to a treatment?
   b) Did it affect your decision to take part in this study? If so why?
   c) Did you have a preference for which trial arm (the intervention or usual care) that you hoped to be assigned to? And why?
   d) We are thinking about running a larger study in the future. How would you feel about joining a study (on the same topic) where you would be allowed to choose which group you were in (intervention or usual care), rather than being randomised?
6. ***For those who dropped out of the study****:* It is helpful to us to understand why people did not complete the trial. Please could you tell me what made you decide to stop taking part? [*Prompt: time commitment, tests, side effects from intervention etc, non-trial reasons]*
7. ***For those who completed the study****:* It is helpful to us to understand why people remain on the trial to completion. Please could you tell me why you committed to the trial to the end?
8. I would like to know your experiences and views about taking part in the trial. Your views will help us to plan our main trial. I’ll ask some specific questions about this.
   a) Overall how did taking part in the trial impact on your daily life?
   b) What was your experience of attending the extra appointments?
   c) Was the frequency and timing of the appointments acceptable, and why?
   d) Would you suggest we change the number and timing of appointments for the main trial and why?
   e) Was there anything else you wanted to say about the appointments?
9. You had to fill out some questionnaires during the trial.
a) Was the time needed to complete these reasonable? And why?
b) Was the frequency of completing them reasonable? And why?
c) What challenges, if any, did you have completing them? *(Prompt: language, understanding, irrelevant etc)*
d) Which of the questionnaires did you feel were relevant to you? And why?
e) Which of the questionnaire did you consider were irrelevant to your experience in the trial? And why?
f) Did you think anything else should be measured that was not included in this trial?
g) For our main trial we want to ensure that we are including measures that are relevant and capture your experiences. Thinking of the questionnaire you completed during the trial, and also thinking about other things you felt were missing, what questionnaires would you suggest that we should definitely include in the main trial?
10. How was the communication between the study team and your usual clinical care team?
11. This trial has been conducted during the Covid-19 pandemic. Did this affect your decision to participate in the trial in any way?
12. Finally, I would like to ask for your views and ideas about how we can recruit other women with SCD in we run a definitive RCT in the future
    a) Thinking back to how you came to hear about the study, can you tell me who first talked to you about it?
    b) Do you have any thoughts about how we approach women initially? What aspects were helpful to help you decide? What aspects were not helpful? Was there anything that could have been improved?
    c) What were your concerns – if you had any - about the study that made you think carefully before you spoke to the research team? How were your concerns allayed? How could we ensure we allay concerns to other women in the future?
    d) What things about taking part in the study worried you and what aspects did not concern you? (prompt extra visits, tests etc.)
    e) How could the doctor have communicated the study better?
    f) Could the research nurse have improved how they explained things? If so what were these, and why?
    g) Do you think the patient information sheet could be improved and how?

**ADDITIONAL QUESTIONS FOR PARTICIPANTS ALLOCATED TO THE INTERVENTION ARM**

13. Please can you tell me about your experience of receiving SPEBT?
14. What were your views on the timing of the blood transfusions? Was every 6-10 weeks acceptable to you? How many transfusions did you have in all?
15. What were your experiences of the blood transfusion you received during the trial? Did you experience any side effects?
    *Have you had transfusions before? If yes, how did this compare?*
16. Is there any aspect about the intervention that you would like to change and why?
17. Has taking part in this study had any impact on your ongoing/future treatment for your condition? If so, how?

Before we end, is there anything else you would like to ask or add to what you have said?

**Debriefing statement**

Thank you very much for all your help today. The team and I really appreciate your time to help us with our research. I would like to reassure you again, that what you have said will remain confidential and will not affect your medical care. When I analyse your data, I will ensure you name and any information that may identify you, will be removed so the transcript of this interview will be completely anonymised. When the trial is complete, we will be writing to everyone who took part in this study to let you know the results and you can also see a summary from all the interviews if you would like.
