# Supplementary material for: The acceptability and feasibility of a randomised controlled trial of serial prophylactic exchange blood transfusion versus usual care in pregnant women with sickle cell disease: a qualitative study
Source: BMC Pregnancy Childbirth. 2026 Jul 3;26:1070. doi: 10.1186/s12884-026-09244-5 (PMC13617929; doi:10.1186/s12884-026-09244-5)
Supplement: Supplementary file 2 [file 12884_2026_9244_MOESM2_ESM.docx]

**<audio recording commences>**

Before we begin, I know you have already provided written informed consent to take part in this interview, I would like to double check you still agree to take part and are happy for me to record this interview. Please could you confirm this again for the recording?

1. Can I just start by asking whether you have you been involved in any research studies before?
2. Thinking back, at what point did you decide not to take part in the trial? [*Prompt: Was it when it was first mentioned to you (before you had much information), when you read the information sheet, or when the research nurse called you?]*
3. At the point at which you made your decision, did you feel you had been given enough information about the study?

*[Prompts: For example, can I just check, were you clear about…What the aim of the trial was? What the intervention was? What taking part in the trial would involve in terms of your commitment?]*

4. What were the reasons why you did not wish to take part in this trial? *[Prompt; practical issues such as time commitment etc; worried about intervention; concerns about impact on child health; concerns about impact on own health; never taken part in research; unclear what was involved, negative views from significant others; being randomized; needed more time to consider]*
   a. This trial is being conducted during the Covid-19 pandemic. Did that affect your decision to take part?
   b. Do you have any mistrust of health professionals? (ethnicity)
   c. Was there anything that prevented you from taking part in this trial? If so, could this have been changed?
5. Thinking back to when you were approached about this research trial, and given some information about taking part, could anything have been improved? *[Prompts: for example…How the research was introduced? The way the information sheet was written? How the clinician and researcher spoke to you?]*
6. We hope that this study will help us improve how the design of future research we might do on this topic. Based on your experience, what suggestions do you have about how we could improve how we get women to join a future research study?
   a. Would you have be more likely to take part if this was a patient preference trial i.e. could decide what you would be allocated to i.e. choosing to have regular blood transfusions during your pregnancy or just staying on your usual care.
   b. If so, what study arm would you have selected and why?
7. Finally, we’d just like to ask you a couple of questions about your history.
   a. Is this your first pregnancy?
   b. In the few years before your pregnancy, how were you with your sickle cell disease? (crises, hospital admissions)
   c. Have you ever had a blood transfusion?
8. Before we finish, is there anything else that you would like to add?
