# Supplementary material for: The acceptability and feasibility of a randomised controlled trial of serial prophylactic exchange blood transfusion versus usual care in pregnant women with sickle cell disease: a qualitative study
Source: BMC Pregnancy Childbirth. 2026 Jul 3;26:1070. doi: 10.1186/s12884-026-09244-5 (PMC13617929; doi:10.1186/s12884-026-09244-5)
Supplement: Supplementary file 3 [file 12884_2026_9244_MOESM3_ESM.docx]

**INTERVIEW QUESTIONS <audio recording commences>**

1. Briefly please can you explain your role in this trial?

Trial Recruitment

2. Do you have any specific feedback on recruitment processes from your perspective?
   a. Overall, what do you think about how many patients were recruited in your site?
   b. How could we improve the way we recruited patients?
   c. How could we better identify patients for the trial?
   d. Looking back, how do you think we could improve communication about the trial? For example, in terms of (a) how patients hear about the trial and (b) how you/other clinicians discuss the trial with them? *[Prompt: content of discussion, approach and/or style of communication).*
   e. Is there anything that would help you to discuss the trial with patients in future: [*Prompt: Would a proforma help? Advice on what to say? How to handle any difficult questions]*

Following the study protocol

3. It would be really helpful to understand your experience of following the study protocol. Specifically,
   a. Did you find any aspect challenging? If so, why?
   b. Was there anything that could be improved, and why?
   c. Were you able to undertake the study as per the protocol? If not, why?
   d. Anything else you particular suggest needs to be changed and why?

Trial intervention

4. Now coming to the actual trial intervention
   a. How did you find delivering the intervention? *[Prompt; time burden, integration into routine practice].*
   b. How could we improve delivery next time?
   c. What is your perception of the effectiveness of this intervention as compared to usual care?
   d. What were your experiences and views about the side effects of this intervention?

Future trial design

5. What are your thoughts about the main trial being a patient preference trial rather than a randomized controlled trial?

How we can improve recruitment for the main trial

6. Do you have any suggestions as to how we can improve the number and rate of recruitment for the main trial?
   a. Would you change the inclusion/exclusion criteria & how and why?
   b. Have you any suggestions on how we can better identify potential patients within the timeframe?
   c. Would you consider extending the recruitment timeframe and if so, why?
   d. Looking back, what were the main reasons why women were not interested in taking part? Is there anything you think we could do to address this in our main trial?
   e. Do you think covid had any impact on whether women signed up for the trial? If so, can you briefly explain?
7. Are there any other relevant points you could like to discuss or mention before we finish?
